# Species differences and human relevance of the toxicity of 4-hydroxyphenylpyruvate dioxygenase (HPPD) inhibitors and a new approach method in vitro for investigation

**DOI:** 10.1007/s00204-023-03458-8

**Published:** 2023-02-17

**Authors:** Jane Botham, Richard W. Lewis, Kim Z. Travis, Audrey Baze, Lysiane Richert, Elizabeth Codrea, Giovanna Semino Beninel, Jean-Christophe Garcin, Christian Strupp

**Affiliations:** 1 Syngenta, Bracknell, UK; 2 Regulatory Science Associates, Inverkip, Scotland, UK; 3 KaLy-Cell, Plobsheim, France; 4 Gowan Company LLC, Yuma, USA; 5 Bayer Crop Science France, Sophia Antipolis, Valbonne, France; 6 Gowan Crop Protection Ltd., Reading, UK

**Keywords:** 4-hydroxyphenylpyruvate dioxygenase, HPPD, Tyrosine aminotransferase, TAT, Human relevance, New approach methods

## Abstract

The mode of action (MoA) of the 4-hydroxyphenylpyruvate dioxygenase (HPPD) inhibitor herbicides in mammals is well described and is generally accepted to be due to a build-up of excess systemic tyrosine which is associated with the range of adverse effects reported in laboratory animals. What is less well accepted is the basis for the marked difference in the effects of HPPD inhibitors that has been observed across experimental species and humans, where some species show significant toxicities whereas in other species exposure causes few effects. The activity of the catabolic enzyme tyrosine aminotransferase (TAT) varies across species including humans and it is hypothesized that this primarily accounts for the different levels of tyrosinemia observed between species and leads to the subsequent differences in toxicity. The previously reported activities of TAT in different species showed large variation, were inconsistent, have methodological uncertainties and could lead to a reasonable challenge to the scientific basis for the species difference in response. To provide clarity, a new method was developed for the simultaneous and systematic measurement of TAT in vitro using robust methodologies in a range of mammalian species including human. The results obtained showed general correlation between high TAT activity and low in vivo toxicity when using a model based on hepatic cytosol and a very convincing correlation when using a primary hepatocyte model. These data fully support the role of TAT in explaining the species differences in toxicity. Moreover, this information should give greater confidence in selecting the most appropriate animal model (the mouse) for human health risk assessment and for key classification and labeling decision-making.

## Introduction

**Fig. 1:**
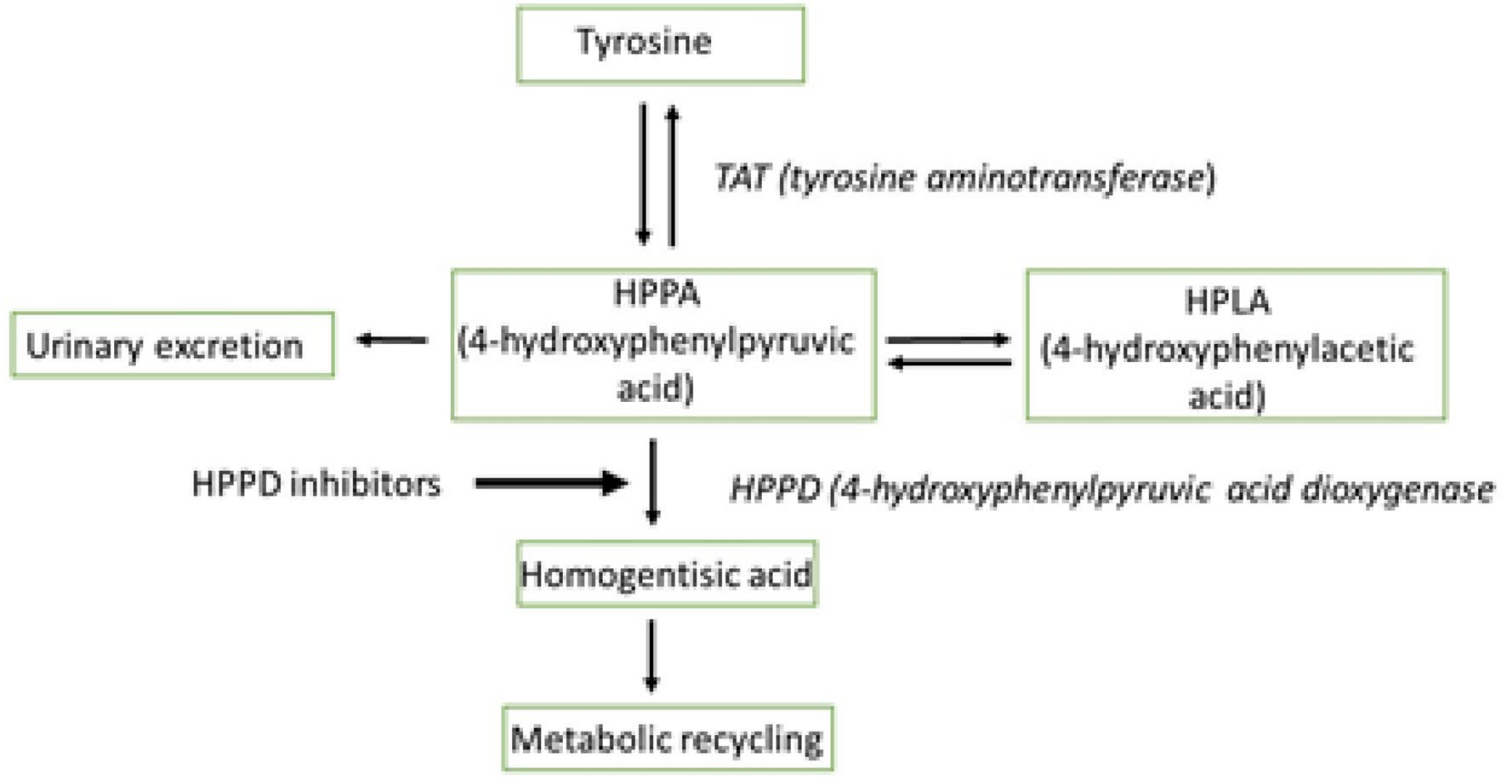
The catabolism of tyrosine in mammals

4-Hydroxyphenylpyruvate dioxygenase (HPPD) is a key enzyme of tyrosine metabolism in plant (and of tyrosine catabolism in animals) and because of their effects in plants, HPPD inhibitors have been successfully developed as agricultural herbicides. The mode of toxic action (MoA) of the HPPD inhibitor herbicides in mammals has been empirically established in experimental animals for a number of chemicals with this herbicidal mode of action. The key events in the MoA involve inhibition of the enzyme HPPD, the second enzyme in the tyrosine catabolic pathway, resulting in excess systemic tyrosine (tyrosinemia). When HPPD is inhibited, the clearance of excess tyrosine is dependent upon catabolism by the first and rate-limiting enzyme in the catabolic pathway, tyrosine aminotransferase (TAT) and elimination of the products via the urine (Fig. 1). The inherent activity of TAT varies across species including humans and it is hypothesized that this primarily accounts for the different levels of tyrosinemia observed between species when given the same amounts of HPPD inhibitor. In rats, for example, TAT activity is considered to be low, and hence at steady state, this species catabolizes tyrosine relatively slowly with the consequence that tyrosine accumulates to very high systemic concentrations, which when maintained for prolonged periods directly results in ocular effects. In addition, there is a positive correlation between a range of other adverse biological endpoints and elevations in plasma tyrosine (Lewis and Botham 2013). The analysis presented in this paper is based on a large database comprising ten agrochemical HPPD inhibitors and one pharmaceutical HPPD inhibitor registered or submitted for future registration. For each of these, there is a full dataset of acute, sub-chronic and chronic toxicity studies across different species. The toxicity of HPPD inhibitors is notable in that model species respond very differently following treatment (Lewis and Botham 2013). Species such as human and the mouse respond much less to HPPD inhibitor exposure and have been reported to have higher levels of TAT activity than species such as the rat which has been reported to have very low levels of TAT activity. For this reason, it is believed that differential TAT levels between species underly this difference in toxicity and species such as the mouse should be considered as more relevant for human health risk assessment than for example the rat which may be considered an inappropriate model (Yozzo and Perron 2020).

Central to confirming (or otherwise) the hypothesis regarding the role of TAT in the species difference in response are accurate data on TAT activity in key experimental species (rat, mouse, dog and rabbit) and human. Existing data on the differences in the occurrence of ocular corneal lesions have been reviewed across the available HPPD inhibitors used as pharmaceuticals and agrochemicals and can be associated with the maximal tyrosine level in each species and inversely related to the reported TAT activity (Table 1).

**Table 1:** Corneal lesions noted in different mammalian species and associated maximal plasma tyrosine concentrations

Species	Rat	Dog	Rabbit	Mouse	Human
Ocular lesions in safety studies*	Consistently seen at very low doses Shape of dose–response curve matches tyrosine, not drug/active ingredient	Sporadically observed	Not observed although the available studies may be too short to confidently conclude absence	Not observed	Clinical observations: only in individuals with severe hereditary tyrosine pathway deficiencies; seldom and fully reversible if dietary protein intake is reduced
Maximum level of tyrosine (nmol/mL) when HPPD fully inhibited	2673 Lock et al (1996)	1814 Roberts (2006) Lock et al. (2006)	1480 Lock et al. (2006)	1154 Duerden 2006) Lock et al. (2000)	1192 Hall et al. (2001)
TAT activity (nmol/min/mg protein)**	1.7 ± 0.2 (males) 3.3 ± 0.5 (females)	13.5 (male) Lock et al. (2006)	3.8 ± 0.85 (females)	7.8 ± 1.5 (males) 10.5 ± 1.9 (females)	~ 7.3 ± 1.17 (male and female)

*Data for up to 10 agrochemicals taken from European Draft Assessment Reports (DARS) in the public domain

**Data taken from Yozzo and Perron (2020) unless otherwise specified

There are several peer reviewed papers (Lock et al 1996, 2000, 2006; Lewis and Botham 2013) and reviews (Yozzo and Perron 2020) where the hepatic activity of TAT (Vmax) has been estimated by the method of Schepartz 1969. These reports provide some limited data across species (sometimes from a single animal in the case of the dog) and indicate a high degree of variability within and, where data are available, across species. Moreover, the detail of the methodology used is sometimes scant as is the provenance of some of the samples assessed.

It is a reasonable assumption that the very different values for the activity of TAT reported may simply be a consequence of the indirect measurement of HPPA in hepatic cytosol, the basis of which is a reaction between aryl pyruvates and phenazine methosulphate (Schepartz 1969).

Analysis of the available data and methodology indicates the potential for cross-reaction with the HPPD inhibitor and/or constituents of the corn oil vehicle (in the case of in vivo studies) as well as any other aryl pyruvates present. In addition, the exact chemical structure of the colored product used for quantitation was unknown but was known to be thermally unstable (Schepartz 1969). Therefore, as a colorimetric assay there is the opportunity for interference from other chemistries and uncertainties over the stability of the complex quantified. In fact, the potent HPPD inhibitor NTBC (2-(4-methylsulfonyl-2-nitrobenzoyl)-1,2-cyclohexanedione) is known to interfere with this assay, perhaps not surprising as NTBC has a very long half-life and is designed to preferentially bind the enzyme HPPD and as such is a mimic for HPPA.

Therefore, care needed to be taken during this spectrophotometric quantitation process adding another potential vulnerability of the practical application of this assay methodology. For these reasons the TAT activity quoted in reports and publications using the Schepartz assay, particularly in the presence of an HPPD Inhibitor with a long biological half-life and/or corn oil vehicle, may be considered of limited reliability and insufficient to fully elucidate the hypothesis that differences in hepatic TAT activity underly the species difference in toxicity.

In view of the methodological concerns raised above and the lack of the systematic generation of TAT activity data in vitro across all species used in safety assessment studies, an approach was devised to address the current variability and uncertainty. A new method has been developed by the Gowan Company where the specific activity of TAT is based upon the direct measurement of 4-hydroxyphenylpyruvic acid (HPPA), the substrate of HPPD. Using this new approach to the measurement of HPPA, per se, the Vmax of TAT has been measured in hepatic cytosol of several animal species and humans. The analyses have been extended by further studies in primary hepatocytes allowing comparison with the Vmax of TAT obtained from preparations of liver cytosol.

The purpose of this paper therefore is to present contemporary in vitro data on hepatic TAT activities from hepatic cytosol fractions and primary hepatocytes across relevant species using a more precise analytical method. An assessment will be made if the data generated to current best scientific standards and methods support the role of differential TAT activity in the species difference in ocular and other toxicity following exposure to HPPD inhibitors and will guide the selection of the most appropriate model species for human health risk assessment and classification purposes.

## Materials and methods

### Analytical method LC–MS

All reagents and solvents used in this study were of analytical grade. The analyte standards, HPLA and HPPA, and internal standard (IS), HPLA-d3, were commercially purchased from Sigma Aldrich.

The method was developed and validated for HPPA and HPLA measurements in hepatocyte cell lysate preparation from 5 different species prepared as described further below as well as in Williams E cell culture medium. Validation samples were prepared with respective incubation matrices mixed with the internal standard solution. Each method validation sample plate was prepared manually and contained two replicates of six calibration standards, six replicates of each quality control (QC) sample, duplicate zero-level standards and duplicate matrix blank samples.

Validation samples were prepared by adding 200 μL of each calibration, QC sample (prepared in respective cell lysate) to 200 μL of IS working solution. The zero standard (no analyte, IS only) was prepared by adding 200 μL of respective blank cell lysate to 200 μL of IS working solution.

The matrix blank samples were prepared by adding 200 μL of respective cell lysate to 200 μL of 90:10 v/v water: ACN (IS solvent). Three batches were prepared for the full validation. The validation plate was vortexed and centrifuged prior to analysis by LC–MS/MS. Dilution linearity was evaluated for each method in respective cell lysate by preparing six replicates of dilution QC samples analyzed as part of a validation batch or injected as a separate batch with calibration standards, batch acceptance QCs and blank samples.

A summary of the chromatographic and LC–MS conditions is provided in the table below.

**Table:** 

Stock solution	2 mM in 50:50 v/v water:acetonitrile
Validated curve range	100–20 000 nM (quadratic 1/x regression)
Vessel type used	Glass
Standard curve concentrations	100, 250, 2 500, 10 000, 15 000 and 20 000 nM
Instrument	Sciex 5500 QTrap
Mobile phase	Mobile phase A: 0.2% v/v Formic acid in water Mobile phase B: 0.2% v/v Formic acid in acetonitrile Rinse: 49:49:2 v/v/v acetonitrile:methanol:formic acid
Solvent flow	0.2 ml/min
Analytical column	Waters Acquity UPLC BEH C18 (1.7 µm, 2.1 × 100 mm) (Guard Column Phenomenex Luna C8)
Analytical run time	~ 9 min
MRM transition (for the analyte and the internal standard)	HPPA: 179.0/107.1 HPLA-d^3^ (IS): 184.0/138.0

A batch consisting of approximately 85 samples was analyzed to demonstrate the stability of prepared samples and method performance over the duration of a typical planned analytical batch.

Validation of the method was carried out according to the FDA bioanalytical method validation guidelines (2018) and SANCO/3030/99 guidance document (rev. 5, 2019) with respect to the linearity, selectivity, precision, accuracy, recovery, limit of detection, limit of quantification, matrix effect and post-preparative stability.

**Table:** 

Method validation results summary	
Intra-batch mean accuracy of calibration standards	95.4–102%
Intra-batch mean accuracy of validation QC samples	96.0–100%
Intra-batch precision (% CV) of validation QC samples	3.0–4.6%
Reinjection reproducibility	5 days
Internal standard selectivity	No peak in the region of internal standard (IS) retention that is > 10% of the mean peak response for all IS responses obtained for the acceptable calibration standards
Analyte carryover	No peak in the region of analyte retention that is greater than the mean response (area ratio) of the acceptable LLOQ standards
Analyte to internal standard selectivity	No peak in the region of IS retention that is > 10% of the mean peak response for all IS responses obtained for the acceptable calibration standards

Based on the validation results, the method was considered fit for purpose

### Measurement of TAT in liver cytosol

#### Materials

All chemicals were obtained from Sigma Aldrich if not indicated otherwise.

#### Test system

Pooled liver cytosols from Wistar rat, C57Bl6 mouse, Beagle dog and human were prepared by KaLy-Cell and pooled liver cytosols from New Zealand rabbit were obtained from Tebu-Bio.

**Table:** 

Species	Gender	Pool size
Wistar rat (*Rattus Norvegicus*)	Male and/or female	6
C57Bl6 mouse (*Mus musculus*)	Male and/or female	6
Beagle dog (*Canis domesticus*)	Male and/or female	6
New Zealand rabbit (*Oryctolagus cuniculus*)	Male	6
Human (*Homo sapiens*)	Male and/or female	6

#### Sample preparations

Cytosols were thawed on ice (0 °C). The protein concentration of the cytosol pools was adjusted to 10 or 20 mg/mL protein concentration. L-tyrosine, 2-oxoglutarate and pyridoxal phosphate were each prepared individually in 0.2 M phosphate buffer in order to obtain working solutions of, respectively, 5.71 mM L-tyrosine, 200 mM 2-oxoglutarate and 0.66 mM pyridoxal phosphate. In a 96-deep well plate sitting on ice, the reaction mixture was prepared by mixing 20 μL of cytosol (0.5 mg/mL, final concentration) with 10 μL of incubation buffer. After a 10 min pre-incubation in a shaking water bath at 37 °C, 170 μL of a pre-incubated at 37 °C mixture composed of L-tyrosine (4 mM, final concentration), pyridoxal phosphate (0.033 mM, final concentration) and 2-oxoglutarate (10 mM, final concentration) was added to start the reaction. At each time-point (0, 30 60 min or 0 and 60 min), the reaction was stopped by adding 200 μL of acetonitrile. Plates were kept at − 80 °C until the shipment in dry-ice to Pharmacelsus GmbH, Saarbruken Germany, for analysis. Samples were analyzed using calibration samples of different HPPA concentrations using individual cytosols as matrix.

### Measurement of TAT in primary hepatocytes

Pooled cryopreserved primary hepatocytes from different species were obtained from Sekisui Xenotech, Kansas City, USA. All experiments were carried out to OECD standards of Good Laboratory Practice.

**Table:** 

Species	Gender	Pool size
Sprague-Dawley rat (*Rattus Norvegicus*)	Male	6
CD-1 mouse (*Mus musculus*)	Male	15
Beagle dog (*Canis domesticus*)	Male	3
New Zealand rabbit (*Oryctolagus cuniculus*)	Male	3
Human (*Homo sapiens*)	Male and female	10

#### Sample preparation

Hepatocytes were thawed in William’s medium E (supplemented with insulin, transferrin, selenium- and linoleic acid, bovine serum albumin, penicillin, streptomycin and tyrosine (100 mg/L)). 300 µL of hepatocyte suspension (10^6^–1.5 × 10^6^ cells/mL) were incubated in triplicate in a 96-well microtiter plate with vehicle control (0.1% dimethyl sulfoxide, DMSO) or NTBC (2 (2 nitro-4-trifluoromethylbenzoyl)-1,3-cyclohexanedione, purity 99.8%) at 100 µM in 0.1% DMSO for 4 h in a humidified culture chamber (37 ± 2 °C, at 95% relative humidity, 95/5% air/CO_2_). Incubation start time was defined by the addition of vehicle or NTBC. At 4 h, incubations were quenched with 600 µL of ice- cold acetonitrile. Samples were centrifuged at 2–8 °C for 5 min at 100 relative centrifugal forces to pellet cells and 100 µL of the supernatant was collected and added to an equal volume of 90:10 acetonitrile: water containing the internal standard (DL-p-hydroxyphenyllactic acid-d^3^; obtained from TCR, North York, Canada) for quantitation of HPPA. This experiment was repeated three times independently for each species.

All standards and quality control samples were spiked with internal standard solution prior to analysis. The presence of HPPA in the cell media supernatant at 4 h was analyzed along with two replicates of six calibration standards and duplicate quality control samples at three different concentration levels using a validated LC–MS/MS method (under good laboratory practice and following the requirements of SANCO 3030/99).

The pelleted hepatocytes were lysed with mammalian protein extraction reagent (300 µL M-PER, Thermo Fisher Scientific) and the protein concentration of each cell lysate sample was determined with a BCA Protein Assay Kit (Thermo Fisher Scientific) against a dilution series of bovine serum albumin. Working reagent was prepared according to the manufacturer’s recommendations and 200 µL was added to 40 µL of cell lysate sample in a 96-well microtiter plate. The microtiter plate was shaken gently for 30 ± 3 min at 37 ± 2 °C. The microtiter plate was allowed to cool, and analysis was conducted at 562 nm with a Synergy HT Multi-Detection Microplate Reader (BioTek Instruments, Inc.). The quantitation of HPPA was normalized to protein content (µmol/mg protein) for each well or tube.

## Results

### TAT measurements in liver cytosol

Preliminary results showed that (i) liver cytosolic basal TAT activity measurement can be performed using substrates of TAT only. This is supported by the observation that HPPA formation in the presence of TAT substrates (L-tyrosine and 2-oxoglutarate) and the co-enzyme pyridoxal phosphate was comparable when ascorbic acid (co-substrate of HPPD) was omitted and when ascorbic acid was present, but HPPD inhibited by NTBC (Table 2), and (ii) HPPA formation was linear up to 60 min at protein concentrations in the incubation medium from 0.1 to 1 mg/mL (data not shown).

**Table 2:** TAT activity (nmol/min/mg cytosolic protein) in the presence and absence of the HPPD inhibitor NTBC

Enzymes/inhibitors	Human*	Dog*	Rat*	Mouse*	Rabbit*
TAT/none	5.53 ± 0.74	0.418 ± 0.020	2.20 ± 0.15	3.80 ± 0.21	2.21 ± 0.32
TAT and HPPD/NTBC	3.75 ± 0.14	0.725 ± 0.055	1.33 ± 0.12	1.92 ± 0.08	1.98 ± 0.18

*Pooled cytosols from 6 animals

To assess the biological reproducibility on liver cytosolic TAT activity, different pools of each species were created and TAT activity was determined using 0.5 mg/mL cytosolic protein, together with substrates of TAT only *(i.e.,* tyrosine, pyridoxal phosphate and oxoglutarate), HPPA concentrations measured twice, with each of the validated standard curves (using mixed and individual cytosols as matrix, respectively), results are given in Table 3. Pool 1 refers to pooled cytosols from 6 experimental subjects of mixed gender, Pool 2 refers to pooled cytosols from 6 males and Pool 3 refers to pooled cytosol samples from 6 females.

**Table 3:** Liver TAT specific activity in three pools of liver cytosols (*n* = 6 subjects) from human, Beagle dog, Wistar rat, C57Bl6 mouse and New Zealand rabbit

TAT activity (nmol/min/mg cytosolic protein)					
0.5 mg/ml cytosolic protein*	Human	Dog	Rat	Mouse	Rabbit
Pool 1	6.92 ± 0.29	0.436 ± 0.017	1.30 ± 0.11	2.49 ± 0.20	2.68 ± 0.77
Pool 2	6.41 ± 0.29	0.645 ± 0.037	1.61 ± 0.18	3.26 ± 0.17	/
Pool 3	5.55 ± 0.32	0.560 ± 0.077	1.18 ± 0.09	2.88 ± 0.12	/
0.5 mg/ml cytosolic protein	Human	Dog	Rat	Mouse	Rabbit
Pool 1	8.25 ± 0.35	0.423 ± 0.046	1.36 ± 0.12	3.29 ± 0.37	3.95 ± 0.56
Pool 2	7.68 ± 0.36	0.715 ± 0.047	1.68 ± 0.18	4.33 ± 0.16	/
Pool 3	6.34 ± 0.44	0.612 ± 0.089	1.26 ± 0.17	4.46 ± 0.34	/

*Validated calibration curve using individual cytosols from each species

**Fig. 2:**
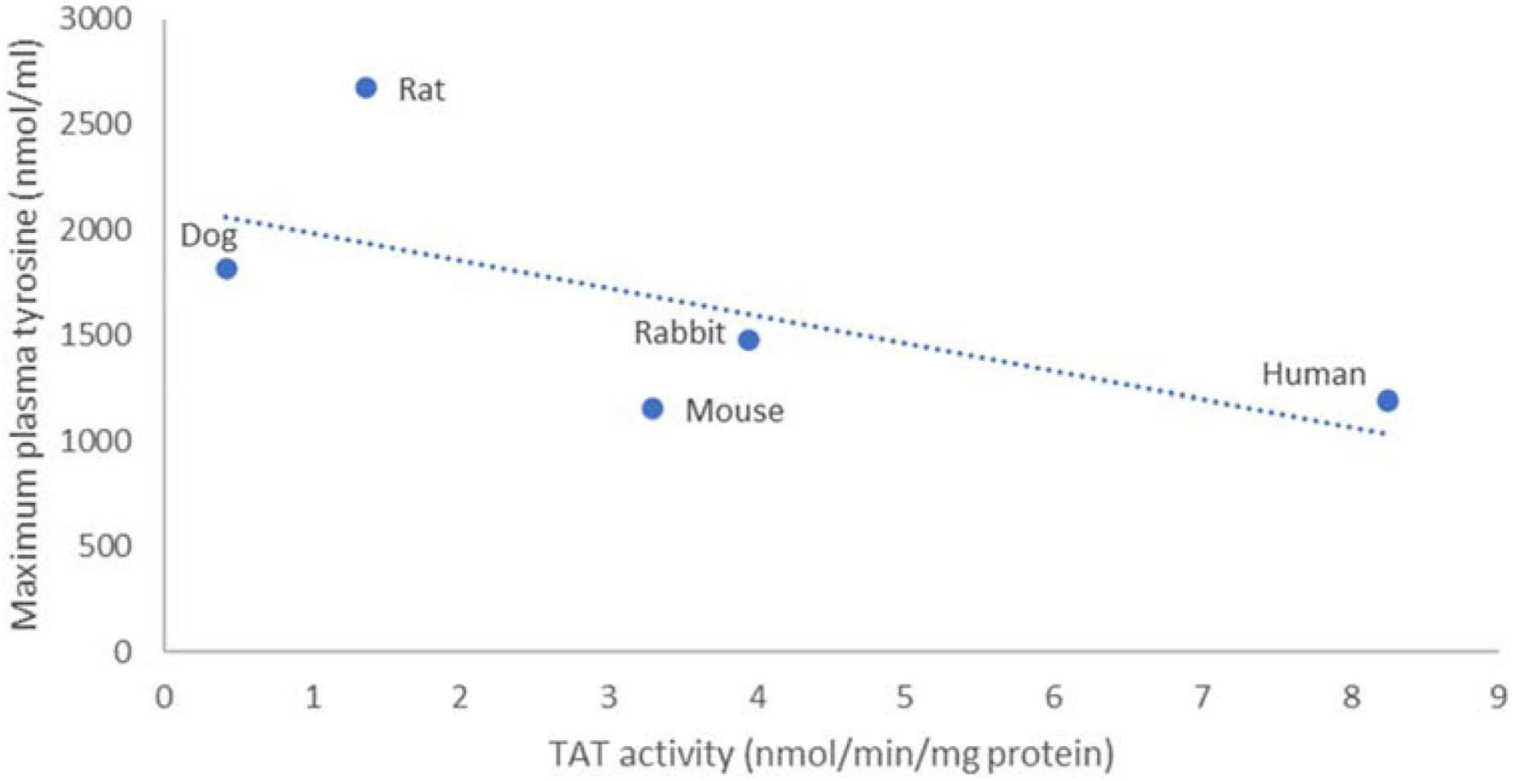
Maximal tyrosinemia and TAT Vmax in cytosol across species

TAT activity in pooled cytosols was Human ≥ Mouse > Rat >  > Dog (see Fig. 2) and no gender effect was observed. More limited data from the rabbit suggest that TAT activity in males would be similar to that of the mouse.

## Primary hepatocytes

The method validated above was used to measure HPPA levels in in vitro cultures of primary hepatocytes from different species. Media were supplemented with 100 mg/ml tyrosine to assure sufficient substrate for formation of HPPA was present and did not become rate-limiting. A series of optimization experiments was performed to assess which culture duration is ideal, and if HPPA is best investigated in cell lysates or culture medium (data not shown). It was concluded that 4 h is an appropriate time-point to have sufficient formation of HPPA for robust detection while integrity of the hepatocyte cultures in suspension without extracellular matrix was still not compromised. Measurement of HPPA in cell lysates had no advantage over measurement in cell culture media, as it was just adding matrix effects in the analytical determination and variability due to slight differences in cell lysis efficiency.

In a definitive proof of concept study, primary hepatocytes from rats, mice, dogs, rabbits and humans were incubated in triplicate with either vehicle control or the pharmacological HPPD inhibitor Nitisinone (NTBC) at a dose completely inhibiting HPPD activity. The study was repeated 3 times independently. Results were as follows (Table 4):

**Table 4:** HPPA formation (TAT activity) in hepatocytes from different species

Species/strain**	Time (h)	Treatment	HPPA (nmol/min/mg protein) ± SD	HPPA (nmol/min/mg protein) Minus vehicle control
Sprague-Dawley rat	4	Vehicle	1.6 ± 0.52	1.4
		NTBC (100uM)	3.0 ± 1.34*	
CD-1 mouse	4	Vehicle	4.4 ± 1.33	19
		NTBC (100uM)	23 ± 5.5*	
Beagle dog	4	Vehicle	0.70 ± 0.146	0.56
		NTBC (100uM)	1.3 ± 0.29	
New Zealand rabbit	4	Vehicle	0.34 ± 0.115	1.0
		NTBC (100uM)	1.4 ± 0.22*	
Human	4	Vehicle	1.9 ± 0.56	16
		NTBC (100uM)	18 ± 6.9*	

*Significantly different from the appropriate vehicle control (0.1%v/v DMSO) as a result of one-way analysis of variance. SD = Standard deviation (study repeated 3 times independently)

**Hepatocytes taken from males with the exception of humans (males and females)

Basal (untreated) HPPA levels in vehicle controls were low and comparable in rats, dogs and rabbits, while humans and mice had slightly higher basal levels in cell culture. This indicates that the activity of TAT is not expressed at the same levels but is different across species. The differences upon full inhibition of HPPD by the pharmacological HPPD inhibitor Nitisinone further elucidated the potential of different species to form HPPA, i.e., to remove excess tyrosine from the system; while in rats and dogs, HPPA levels only rose by less than twofold (+ 75 and + 50%, respectively), they rose approximately threefold in rabbits, fivefold in mice and 8.6-fold in humans within 4 h.

## Discussion and conclusions

As indicated earlier, in the published literature, the reported activities of TAT in different species based on the colorimetric assay of (Schepartz (1969) can show large variation, can be inconsistent, have methodological uncertainties and can lead to reasonable challenge to the scientific basis for the species difference in response to HPPD inhibitors. To provide clarity, approaches were developed for the simultaneous and systematic measurement of TAT in vitro using robust methodologies in a range of mammalian species including human.

To assess if the data generated in liver cytosol fractions are consistent with the hypothesis that differences in TAT activity can explain the species differences in the ocular toxicity of HPPD inhibitors, TAT activities from this in vitro system were compared to the maximal tyrosine levels generated across species in in vivo systems (Fig. 2).

**Fig. 3:**
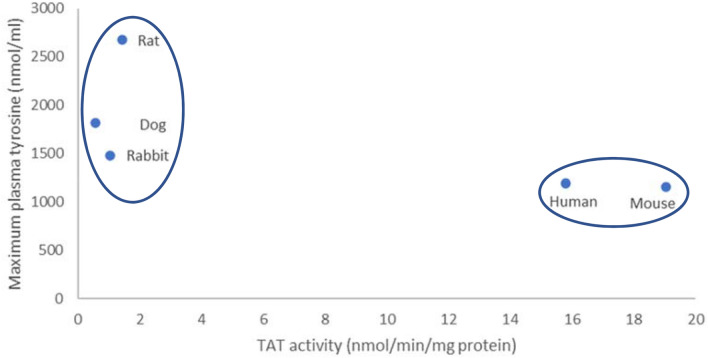
Maximal tyrosinemia (NTBC) and TAT rate in primary hepatocytes across species

On the Y axis of this plot is the maximal tyrosinemia achieved in each species when HPPD is inhibited (data shown in Table 1) and on the X axis is the TAT activity from liver cytosol of that species, quantified by LCMS. Data plotted are taken from Table 3; validated calibration curve using individual cytosols from each species as matrix and Pool 1 (mixed gender). Although there appears to be a reasonable correlation and the data are generally supportive of the ‘TAT’ hypothesis there are some mismatches in that the species with the lowest TAT activity, the dog, although having high levels of tyrosine does not develop the highest tyrosinemia of the species tested. The human seems to have a greater TAT activity than mouse but experiences a similar tyrosinemia. Hence, the data from a subcellular fraction do not fully explain the toxicological findings. Neve et al (2003) reported that HPPD specifically localizes in the endoplasmic reticulum and the Golgi apparatus rather than in the cytoplasm suggesting that analysis of cytoplasm alone may fail to identify true TAT activity. Differences in transport mechanisms across the cell membrane or differences in relative TAT amount per cytosolic total protein are likely affecting and distorting the correlation. When the data from the primary hepatocyte culture model are compared to the same in vivo data on tyrosine levels, a much clearer picture, also matching the toxicities seen in the respective species, is seen (Fig. 3).

In this model, hepatocytes (not hepatic cytosol) are used to mimic in vivo kinetics of tyrosine catabolism to HPPA and with LCMS detection of HPPA/HPLA (instead of the indirect colorimetric assay of Schepartz 1969). The data essentially fall into 2 groups; those with lower TAT activities (rats, dogs and rabbits) develop a significant (> 1000 nmol/ml) tyrosinemia when HPPD is inhibited whereas those with the highest TAT activities (mice and humans) form a second group with a significantly reduced (< 1000 nmol/ml) tyrosinemia induced when HPPD is inhibited. The hepatocyte model offers a reliable and reproducible prediction of the maximal tyrosinemia induced when HPPD is inhibited and the in vitro determination from all species represented now faithfully predict the in vivo response.

In summary, this paper reports the first robust, systematic and simultaneous generation of TAT activity data across species. This enables species comparisons to be made free of the concerns and uncertainties of the historic literature reports where the methodology was suspect and some of the samples evaluated of unknown provenance. Of the two in vitro systems evaluated, the primary hepatocyte model (closer to the in vivo situation) better predicts the observed in vivo responses (tyrosinemia) and hence ocular toxicity, with the results obtained showing a general correlation between high TAT activity and low in vivo toxicity when using a model based on hepatic cytosol but a very convincing correlation when a primary hepatocyte model was used. These findings help to resolve many of the concerns and uncertainties over aspects of the existing database (such as the corneal findings seen sporadically in the dog and their likely relationship to high plasma tyrosine/low TAT activity). In conclusion the work presented fully supports the hypothesis that differences in hepatic TAT activity is responsible for the species difference in toxicity that is a feature of this MoA class and confirms species such as the mouse to be more relevant for predicting effects in humans. A new approach method for direct determination of hepatic TAT activity in vitro by measuring formation of the phenolic acid HPPA was successfully established and validated. This information should give greater confidence in selecting the most appropriate animal model (the mouse) for human health risk assessment and for key classification and labeling decision-making.

## Data Availability

The data on which this work is based are located in the archives of Bayer SAS and Gowan and KaLy-Cell Companies.
